# Unintentional childhood injury patterns, odds, and outcomes in Kampala City: an analysis of surveillance data from the National Pediatric Emergency Unit

**DOI:** 10.5249/jivr.v3i1.56

**Published:** 2011-01

**Authors:** Milton Mutto, Stephen Lawoko, Catherine Nansamba, Emilio Ovuga, Leif Svanstrom

**Affiliations:** ^*a*^Pincer Group International Ltd & Karolinska Institutet Department of Public Health Sciences,Social Medicine,Sweden.; ^*b*^Karolinska Institutet, Department of Public Health Sciences, Social Medicine, 171 76 Stockholm, Sweden.; ^*c*^PO Box 25411, Kampala, Uganda.; ^*d*^Department of Medicine, Gulu University, Gulu, Uganda.

## Abstract

**Background:**

Unintentional Childhood Injuries pose a major public health challenge in Africa and Uganda. Previous estimates of the problem may have underestimated the childhood problem. We set to determine unintentional childhood injury pattern, odds, and outcomes at the National Paediatric Emergency unit in Kampala city using surveillance data.

**Methods:**

Incident proportions, odds and proportional rates were calculated and used to determine unintentional injury patterns across childhood (1-12 years).

**Results:**

A total of 556 cases recorded between January and May 2008 were analyzed: majority had been transported to hospital by mothers using mini-buses, private cars, and motorcycles. Median distance from injury location to hospital was 5 km. Homes, roads, and schools were leading injury locations. Males constituted 60% of the cases. Play and daily living activities were commonest injury time activities. Falls, burns and traffic accounted for 70.5% of unintentional childhood injuries. Burns, open wounds, fractures were commonest injury types. Motorcycles, buses and passenger-cars caused most crashes. Play grounds, furniture, stairs and trees were commonest source of falls. Most burn injuries were caused by liquids, fires and hot objects. 43.8% of cases were admitted. 30% were discharged without disability; 10%, were disabled; 1%, died. Injury odds and proportional incidence rates varied with age, place and cause. Poisoning and drowning were rare. Local pediatric injury priorities should include home, road and school safety.

**Conclusions:**

Unintentional injuries are common causes of hospital visit by children under 13 years especially boys. Homes, roads and educational facilities are commonest unintentional injury sites. Significant age and gender differences exist in intentional injury causation, characteristics and outcomes. In its current form, our surveillance system seems inefficient in capturing poisoning and drowning. The local prevention priorities could include home, road and school safety; especially dissemination and uptake of proven interventions. Burns should be focus of domestic injury prevention among under-fives. Commercial passenger motorcycles require better regulation and control.

## Introduction

Unintentional injuries remain a major global health problem.^1^At least 90% of global injury mortality and 113 million (70 %) injury DALYS are attributed to them. Nineteen percent (19) % of the global [unintentional injury] burden is among children and adolescents^2,3^with road traffic, drowning, burns, falls, and poisonings as leading causes.^2,3,4,5^Drowning, burns and poisoning are particularly problematic in childhood and old age because of associated [high] fatality rates, [limited] treatment successes^6,7^and risks of life-long scars and disabilities.^8,9^Although known to disproportionately (98%) burden developing countries,^4,5^the actual childhood proportion of the [unintentional injury] burden in these countries remains unclear.

In Africa and Uganda, injuries rank among top ten mortality causes.^10,11,12,13^ However, most previous [Ugandan] studies were limited in [geographic] scope, and biased towards severe incidents among older persons. Because of location at the main accident and emergency units, most of these studies under sampled [childhood] injuries, especially poisonings. The exact extent of this under sampling is not, however, clear. Currently, many of the childhood emergencies report directly to the specialized pediatric emergency unit which is located in another part of the national referral hospital (approximately 200 meters from the main accident and emergency unit). Childhood trauma cases presenting at this unit are then referred to the main accident and emergency unit. Poisoning and other medical emergencies are managed directly at the pediatric emergency unit.

In addition, many of the previous studies also grouped ages 1-5 and/ or 1-10 in analysis;^11,12,13^ and yet age specific developmental risks are recognized.^14,15,16^For example, lower cognitive capacities have been associated with certain injury types. It was not clear if [unintentional] childhood injury patterns, odds and outcomes at the pediatric emergency unit differed from previous descriptions and what programmatic and policy implications of such differences would be. We set to describe unintentional childhood injury patterns, outcomes, and odds at the National Pediatric Emergency unit in Kampala.

## Methods

**Study design**

A cross sectional analysis of data collected from all children below 13 years accessing injury care at the National paediatric emergency unit in Kampala between January and May 2008 was undertaken.

**Setting**

Injury surveillance was set-up at the National Referral Paediatric unit, a tertiary level teaching facility in Kampala. Uganda operates a tiered health system with 2 National and 11 Regional Referral facilities. Smaller units are organized under district health services.^17^This facility has total bed capacity of over 1500. Although at the peak of the health care system, it also offers primary care to the city residents. The hospital has a specialized pediatric emergency unit which handles all emergencies except trauma among under-12 year olds. Poisoning cases are handled at the pediatric emergency unit as well. The rest of the traumatic emergencies are referred to and managed at the general accident and emergency unit. Uganda lacks comprehensive pre-hospital services including public ambulances.  The hospital was purposively selected because it already had ongoing injury surveillance at the main accident and emergency unit.

**Population**

Targeted population was unintentionally injured children [below 13 years of age] whose injuries were severe enough to cause health seeking at this or similar facilities. Accessible population was those seeking care at the National referral Pediatric Emergency unit and were captured in the registry: eligibility was restricted to incident cases. The study was cleared by the National Council of Science and Technology and the Ethics committee of the Hospital (UNCST-Ref: HS 226). Adult care takers of the injured children gave consent for participation in the study.

**Data sources and variables**

A trauma registry was established at the pediatric emergency unit to provide primary data. The registry had been set up to pilot childhood injury surveillance using instruments earlier adapted by Adnan etal.^18^All children below 13 years accessing care for unintentional injuries were eligible. Consenting parents provided information to emergency care nurses who completed a form for each injured child. Injury classification was based on ICD-10: analyzed variables included age, gender, time, place, and activity at injury time, intent, injury mechanism, severity, nature, affected body part, and outcomes. Although we did not evaluate the sensitivity of the current registry, a 2000 evaluation estimated the sensitivity of a similar registry at the nearby (main) accident and emergency unit to be 28% with good daytime but poor nighttime coverage.

**Statistical methods**

Data  analysis  was  conducted  using  STATA  8  (College Station, Texas, USA): incident characteristics were disaggreg-ated by age, location and cause. Proportions, odds and proportional incidence were computed and used to determine unintentional injury distribution, patterns, and trends by cause and location across childhood.

## Results

A total of 556 patients were registered between January and May 2008: 60.32 % of them, male. About half (47%) were under-five years old, 84% were transported to hospital by family, (62.5% by mothers, 16.7% by fathers and 5.6% by other family members), 3.2% by teachers and friends. Median distance between injury site and hospital was 5 km. Public taxis were mostly used (47%), followed by private cars (21%), motorcycles (16%) and ambulances (4.6%).

**Place of injury**

Homes accounted for 54.8% of unintentional childhood injury locations: roads constituted 28.9% and schools, 8.4%. Mean ages among accidental home, road and school injured children were 3.8 (SD=2.8), 6.4, (SD=2.9) and 6.8 (SD=2.2). Boys constituted 61% of accidental home injury victims. Commonest activities at home injury times were play (71.2%) and daily living activities (12.1%). Falls constituted 33.4%, burns, 30% and poisoning, 1.2% of accidental home injury causes, while burns (37.7%), cuts/open wounds (24.5%), and fractures (15.2%) were the commonest injury types. Accidental home injury odds were highest in first year of life (seeFigure 1), mostly due to burns and falls. After adjusting for gender and location, injury odds significantly varied with age (Odd Ratio=0.76, CI=0.71-0.81; Odds Ratio=1.2, CI=1.2-1.3; and Odds Ratio=1.2, CI=1.1-1.4 respectively).

**Figure 1:Odds of childhood injury by place and age:**
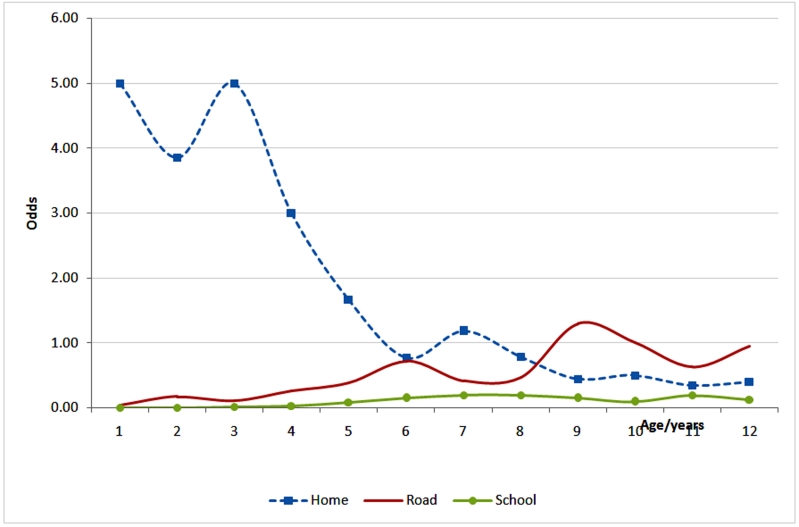


**Activity at incident time**

Commonest unintentional injury time activities were leisure/play (54.6%) and travel (15.6 %). Mean age of accidental play injury victims was 4.5 (SD=2.9), and for travel was 6.1, (SD= 3.1). Play accounted for 20% of activities at traffic injury time. Boys, compared to girls, had excess injury risk of 1.6 in both activities. Falls constituted 38.2%, burns 20.4%, motor vehicle crashes 2.0% and poisoning 0.7% of play injuries.

**Cause of injury**

Burns (17.9%), traffic (25.3%), and falls (27.3%) were leading unintentional injury causes. Majority (73.5%) of traffic injuries occurred to children while walking and 12.5-17.5 % while traveling as car and motorcycle passengers: 22.5% traffic injuries occurred at boarding time. Motorcycles (31.0%), buses/vans (21.6%), and passenger cars (19.6%) were commonest striking objects. Falls constituted 27.3% of unintentional childhood injuries: play ground falls constituted 21.9%, beds, sofas and other home furniture 14.6%, steps/ladders, 10.9% and trees, 10.4% of falls. Burns accounted for 17.9% of unintentional childhood injuries (hot liquids=67.8%, fires=11.8% and hot objects=3.4%). Only 5 cases of mainly (67%) paraffin related poisoning were recorded. Other injuries were dog bites (9.2%), and cuts (6.8%).

Odds of burns were highest in the first year of life, dropping progressively, before stabilizing at age 6 years (seeFigure 2). Odds of traffic and falls gained with age (seeFigure 2below). After adjusting for gender and cause, injury odds significantly varied with age (Odds Ratios=1.2, CI=1.1- 1.24, and 0.6, CI=0.57-0.71, respectively). The effect of age on fall injuries was marginal (Odds Ration=1.1, CI=1.0-1.16).

**Figure 2:Crude odds of injury by age and cause for top three childhood injury causes:**
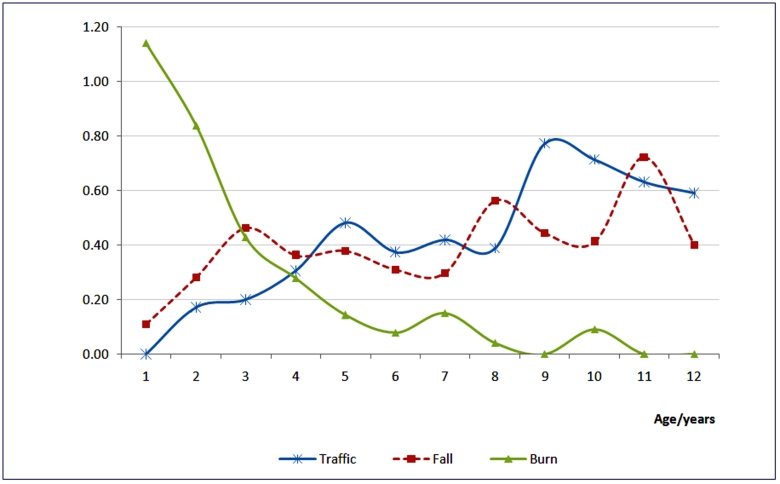


**Nature of injury**

Cuts, bites or open wounds constituted 23.9% of unintentional childhood injuries:  20.7% were fractures, 18.4% were burns and 9.4% were bruises and superficial injuries.  Use of prevention interventions previously shown to be effective varied between zero and 34.5% (supervision of bath /swimming =34.5%, safe hazard storage=17.24 %, seatbelt use= 1.26%, helmets and car air bag use each 0.18% and child car seat use=zero). Majority (93%) of injuries were treated on public welfare (free care by government): only 3%, on insurance.  Up to 43.8% were admitted (see Table 1); 38.6% of patients were discharged without disability, 11.5% with disability. Less than 1 % were in Intensive Care (ICU), 0.2% died at casualty (see Table 2).

**Table:** Table 1:**Severity by anatomical site **

Anatomical site	Minor	Moderate	Severe but not life threatening	Life threatening but survival likely	Critical with uncertain survival	Fatal

Face(N=122)	11.03	7.03	3.01	0.18	0.36	0
Head/neck(N=139)	10.27	8.47	5.23	0.72	0.36	0
Chest(N=80)	3.78	6.49	3.6	0.36	0.18	0
Abdomen(N=90)	7.21	5.41	2.52	0.9	0.18	0
Extremities(N=329)	23.42	19.82	14.05	1.26	0.54	0.18

**Table:** Table 2:**Unintentional incident characteristics by cause**

Cause	Proportion of unintentional injury/%(N=557)	Mean age(sd)	Sex ratio *	Admissions/%	Emergency surgery & ICU/%	Discharge with disability/%

Traffic	25.3	6.11(2.9)	135	59.6	1.40	32.6
Falls	27.3	5.41(3.1)	253	29.6	0.70	42.8
Burns	17.9	2.29(2.1)	122	85.0	-	14.0
Poison	0.90	4.60(4.5)	066	60.0	20.0	-

* Number of males per 100 females

## Discussion

The study described unintentional childhood injury patterns, odds, and outcomes at the National Pediatric unit in Kampala, Uganda. It found unintentional injuries a common cause of hospital visits by children below 13 years of age; most being transported to hospital by mothers using public transport [including commercial motorcycles]. Homes, roads and school facilities were the commonest injury sites; most happening during play or daily life activities. Falls, traffic, and burns were lead unintentional injury causes.  Injury odds varied with age, cause and location. Odds of [unintentional] injury were highest below five, with domestic burns as most likely. Poisonings were rare but largely severe. Boys were one and half times more likely to sustain accidental injury compared to girls: gender differences were highest in falls. Half of patients were admitted; a tenth were disabled: case fatality was low. Admission was most likely in burns; hospitalization for intensive care or emergency surgery was most likely in traffic and poisoning. Disability was most likely in falls and traffic injuries.

Our findings are more reflective of unintentional injury patterns and risks among under-13s in Kampala city than previously described given our data source. We show higher unintentional injury prevalence among under-fives than previously reported: 5-14 year olds were earlier identified as most at risk.^12^Overall; falls were the leading unintentional injury cause among under-13s while burns led below 5. We also show a triple injury burden at age four where burns, falls and traffic approximated parity. Reasons for this were not immediately clear, but developmental correlates could be responsible. Under-fives injury prevention priorities should therefore include domestic burns on account of burden. The two peaks in the distribution of unintentional [childhood] injuries [at ages seven (grade three) and 11 years (grade six)] were traffic and falls related. It was not clear if they had specific developmental and childcare correlates and underpinnings [including parental supervision]; but age 11 heralds puberty. Later childhood injury prevention must address traffic and falls at roads, homes and schools.

We did not detect drowning, although it was previously described as a leading global killer.^2,11^Falls were the leading unintentional injury causes ahead of traffic; (previously, the traffic proportion of injuries was estimated at double the sum total of falls and burns).^13^Our findings confirm: roads, homes and schools as lead locations of unintentional childhood injury ^11^with a male dominance.

A number of factors could account for our findings: firstly the high prevalence of unintentional injuries below age 13 years could have been attributed to greater visibility occasioned upon current registry location at the pediatric unit: previously the prevalence was lower,^12^lending credence to possible bias in previous estimates occasioned upon registry location at general emergency units. Secondly, the observed dominant patient transport mode may have reflected the lack of public ambulance services in Kampala than choice; thirdly, over representation of women in transportation of injured children to hospital could reflect cultural imperatives in child care practices in Kampala which usually assign such roles to women; and finally, the age specific differences in [unintentional] injury odds could be developmental as this influences where children are in time and space, what risks they get exposed to, and the physical, psychosocial and cognitive competencies they possess. The low prevalence of drowning could reflect access limitations to swimming pools, lakes or rivers in Kampala rather than case fatalities, or lack of vital registration. Under-representation of poisoning seemed more of a triage problem given that poison cases are triaged to medical rather than the surgical side where the registry is located. Moreover, childhood poison emergencies are usually managed at the pediatric emergency unit while all other trauma is referred to general accident and emergency unit. We did not, however, assess the actual number of triage related misses.

The big number of motorcycle related injuries could reflect the growing importance of passenger motorcycle services in Uganda and their growing role as lead traffic injury causes. Although convenient, Ugandan passenger motorcycle services are poorly regulated and unsafe. The high burden of [unintentional] childhood burns could reflect persistence of unsafe energy in Ugandan households.^19^Future research in this area could also address safer alternatives for low income communities such as in Kampala. Lack of safe play areas manifested in of the high number of leisure related unintentional childhood injuries. Mabel et at^20^previously showed Kampala schools to be unsafe. The study also found use of interventions previously found effective in preventing accidental injuries to be low. It was not clear if this was a knowledge, attitudes or practice gap among Kampala communities, or an access problem.

Key weaknesses include use of surveillance data: such data are prone to coverage and completeness errors: a similar registry at the main emergency unit was earlier shown to have a sensitivity of 28%. It was not clear if this was still the case with the current registry. Our data may also be more reflective of severe cases. Multiple injuries may not have been appropriately tracked. Our analysis of injury odds was based on artificial cohorts: different risk patterns may have emerged from true birth cohorts of the different ages analyzed. The findings, however, lend further justification for restructuring Uganda’s unintentional childhood injury prevention and research priorities.

## Conclusion

Unintentional injuries are common causes of hospital visit by children under 13 years especially boys. Homes, roads and schools are commonest unintentional injury sites. Significant age and gender differences exist in intentional injury causation, characteristics and outcomes. In its current form, our surveillance system seems inefficient in capturing poisoning and drowning.

The local prevention priorities could include home, road and school safety; especially dissemination and uptake of proven interventions. Burns should be focus of domestic injury prevention among under-fives. Commercial passenger motorcycles require better regulation and control. Active surveillance of unintentional childhood injuries is also recommended.
